# Acetyl-CoA synthetase is activated as part of the PDH-bypass in the oleaginous green alga *Chlorella desiccata*

**DOI:** 10.1093/jxb/erv424

**Published:** 2015-09-10

**Authors:** Omri Avidan, Uri Pick

**Affiliations:** Department of Biological Chemistry, The Weizmann institute of Science, Rehovot 76100, Israel

**Keywords:** Acetyl-CoA synthase, ATP citrate lyase, *Chlorella desiccata*, green algae, PDH-bypass, triacylglycerol biosynthesis.

## Abstract

In a recent study, it has been shown that biosynthesis of triacylglycerol (TAG) in the oleaginous green alga *Chlorella desiccata* is preceded by a large increase in acetyl-coenzyme A (Ac-CoA) levels and by upregulation of plastidic pyruvate dehydrogenase (ptPDH). It was proposed that the capacity to accumulate high TAG critically depends on enhanced production of Ac-CoA. In this study, two alternative Ac-CoA producers—plastidic Ac-CoA synthase (ptACS) and ATP citrate lyase (ACL)—are shown to be upregulated prior to TAG accumulation under nitrogen deprivation in the oleaginous species *C. desiccata*, but not in the moderate TAG accumulators *Dunaliella tertiolecta* and *Chlamydomonas reinhardtii*. Measurements of endogenous acetate production and of radiolabelled acetate incorporation into lipids are consistent with the upregulation of ptACS, but suggest that its contribution to the overall TAG biosynthesis is negligible. Induction of ACS and production of endogenous acetate are correlated with activation of alcohol dehydrogenase, suggesting that the upregulation of ptACS is associated with activation of PDH-bypass in *C. desiccata*. It is proposed that activation of the PDH-bypass in *C. desiccata* is needed to enable a high rate of lipid biosynthesis under nitrogen deprivation by controlling the level of pyruvate reaching ptPHD and/or mtPDH. This may be an important parameter for massive TAG accumulation in microalgae.

## Highlight

Plastidic acetyl-CoA synthase (ptACS-2) and ATP citrate lyase are upregulated in the oleaginous alga *Chlorella desiccata* during nitrogen deprivation. ptACS-2 is part of the pyruvate dehydrogenase bypass which supports triacylglycerol accumulation.

## Introduction

Many green algae accumulate, under growth limiting conditions—such as nitrogen deprivation, large quantities of storage products such as starch or triacylglycerol (TAG). However, the regulatory constrains controlling carbon flow towards starch or TAG synthesis are poorly understood. Two transcription factors that seem to control TAG accumulation in *Chlamydomonas reinhardtii* have been identified: *Nrr1*, which is required for lipid accumulation only under nitrogen deprivation, and has not been identified in lipid-accumulating microalgae species (Boyle *et al.*, 2012); and the recently identified PSR1, which appears to function as a global regulator of TAG accumulation under different nutrient limitation conditions (Ngan *et al.*, 2015). Recent reports have linked between the availability of carbon precursors that can be utilized for fatty acids (FA) biosynthesis—such as acetate or pyruvate—and the rate of TAG accumulation in plants and algae (Goodson *et al.*, 2011; Siaut *et al.*, 2011; Fan *et al.*, 2012; Ramanan *et al.*, 2013; Goodenough *et al.*, 2014), implying that carbon flux is a major determinant controlling the rate of lipid synthesis and accumulation in these species. Interestingly, the TAG accumulation capacity, commonly achieved by nutrient limitations such as nitrogen, varies remarkably between species, including green microalgae (Rodolfi *et al.*, 2009; Dean *et al.*, 2010; Yeh and Chang, 2011; Praveenkumar *et al.*, 2012; Schuhmann *et al.*, 2014). Based on such studies, it has been proposed that the ability of cells to divert their cellular carbon and nitrogen metabolism, is a major means to cope with N limitations and an important factor affecting TAG accumulation capacity of each species (Goodenough *et al.*, 2014; Li *et al.*, 2014; Schmollinger *et al.*, 2014). In a recent study, the authors demonstrated that the steady-state levels of Ac-CoA remarkably increase prior to the accumulation of TAG in N-deprived green microalgae and that they were substantially higher in the high TAG-accumulating alga, *Chlorella desiccata* (Avidan *et al.*, 2015). Moreover, the calculated cellular concentrations of Ac-CoA were far lower than reported *K*m_Ac-CoA_ values of plastidic acetyl-CoA carboxylase in plants. It was thus suggested that the capacity to accumulate high levels of TAG in algae critically depends on their ability to divert carbon flow towards the synthesis of Ac-CoA following N limitation, consistent with the idea that carbon flux is a major determinant controlling TAG accumulation.

Different Ac-CoA producing enzymes have been previously associated with the regulation of FA biosynthesis in various organisms. In animals and yeast, in which FA biosynthesis is cytosolic, the major Ac-CoA producer is ATP citrate lyase (ACL), which is recognized as the rate-limiting enzyme for lipogenesis and lipid accumulation (Boulton and Ratledge, 1981; Ratledge and Wynn, 2002; Ma *et al.*, 2009). Although in plants ACL was suggested to be involved mainly in protein acetylation, flavonoids biosynthesis, and FA elongation, *acl* mutant *Arabidopsis thaliana* exhibit dramatic abnormalities, indicating that ACL is required for normal growth and development also in plants and that no other source can compensate for cytosolic ACL-derived Ac-CoA (Fatland *et al.*, 2002, 2005; Beisson *et al.*, 2003).

In plants and algae, the major chloroplastic Ac-CoA producer is plastidic pyruvate dehydrogenase (ptPDH), which was demonstrated to be a critical enzyme for FA biosynthesis in autotrophic species (Shtaida *et al.*, 2014). In a previous study, the authors have shown that ptPDH was upregulated in correlation to Ac-CoA elevation in an oleaginous green microalga species during N limitation, consistent with the idea that it is the major supply of Ac-CoA in this species (Avidan *et al.*, 2015).

Nevertheless, the first enzyme implicated in plastidic Ac-CoA biosynthesis in plants was Ac-CoA synthetase (ACS2), which converts aerobic fermentation-derived acetate into Ac-CoA (Roughan and Ohlrogge, 1996). Later works, however, have shown that in developing *Arabidopsis* seeds there is insufficient acetate to account for the rate of FA synthesis and that its temporal and spatial mRNA expression are not correlated with TAG accumulation (Bao *et al.*, 2000; Ke *et al.*, 2000). In the green algae *C. reinhardtii*, ACS-2 and ACS-3 are upregulated during an acetate boost which increases TAG biosynthesis, both in the wild-type and in the TAG-accumulating *sta6* strains, implying that these enzymes are involved in channelling acetate into FA and TAG (Goodenough *et al.*, 2014).

In this study, the expression of plastidic ACS2 and ACL was followed in response to N deprivation in three green microalgae species that were compared in a previous study (Avidan *et al.*, 2015): *C. desiccata*, a high TAG accumulator, and two moderate TAG accumulators, *Dunaliella tertiolecta* and *C. reinhardtii*. Their expression was compared with that of other Ac-CoA producers and it is shown that ACS and ACL are upregulated only in the high TAG-accumulating alga *C. desiccata*. Moreover, evidence is provided that ACS2 is induced as part of the PDH-bypass, possibly as a mechanism to cope with N deficiency.

## Materials and methods

### Algal strains and cultivation conditions

*D. tertiolecta* was obtained from the culture collection of Dr W. H. Thomas (La Jolla, CA); *C. reinhardtii cw15* was obtained from Prof. A. Danon (Department of Plant Sciences at the Weizmann Institute); and *C. desiccata* (UTEXID LB2437) was obtained from The Algae Culture Collection at the University of Texas at Austin.

Cells were grown under continuous illumination (400 µmol m^–2^ s^–1^) at 24 °C in either artificial sea water medium (ASW, modified f/2 medium, Guillard and Ryther, 1962) containing 10mM nitrate and bubbled with air enriched with 5% CO_2_ (*C. desiccata*), TAP medium (Gorman and Levine, 1965) containing 7mM ammonium (*cw15*), or in 2M NaCl Dunaliella medium containing 5mM nitrate and 50mM NaHCO_3_ as previously described (Rachutin-Zalogin and Pick, 2014*a*). In order to induce triglyceride accumulation, mid-log-phase cells were washed and transferred to a nitrogen-depleted (–N) medium at the following initial concentrations: *C. desiccata* 2×10^7^ cells/ml, *D. tertiolecta* 2×10^6^ cells/ml, and *cw15* 1.5×10^6^ cells/ml.

### Chlorophyll measurements

Cell pellets (1ml culture sample) were suspended in 80% acetone and collected by centrifugation. For *C. desiccata*, cell pellets were first permeabilized by pretreatment with 50 µl DMSO at 70 °C for 5min followed by the addition of 80% acetone (1ml final volume). The absorbance of the extracts was measured at 663.6nm and 646.6nm and chlorophyll content was calculated as previously described (Porra, 2002).

### Triglyceride analysis by TLC

For TLC, lipids were extracted by the following method: pellets of 1–5×10^7^ cells were suspended with 200 μl of DMSO, heated to 70 °C for 5min, mixed (vortex) with 3ml MeOH, and left for 12h at 4 °C. Cell pellets were collected by centrifugation and saved for starch analysis, while the remaining supernatant was mixed vigorously with 3ml diethylether, 3ml *n*-hexane, and 3ml double-distilled water before centrifugation for 5min at 3200 *g*. The upper *n*-hexane phase was separated and evaporated in a desiccator; dried lipids were resuspended with 200 μl chloroform and kept at –20 °C. One to two microlitres was applied to TLC silica-gel plates (5×7.5cm, 60 F254, Merck), and developed in a closed jar in a mixture of *n*-hexane:diethylether:acetic acid (85:15:1, v/v/v). Lipid spots were visualized by 5min incubation in iodine vapour. The plate was scanned by an Image Scanner III, Epson ExpressionTM 10000 XL using scanning software LabScan™ 6.0 (Powered by Melanie, Swiss Institute of Bioinformatics). TAG levels were quantified by densitometry software ImageQuant™ TL relative to different amounts of Triolein standards.

### Gene expression analysis

For RNA purification, collected samples were treated with TRI reagent, according to the manufacturer’s protocol (Molecular Research Center). Complementary DNA was synthesized by qScript cDNA Synthesis Kit (Quanta), using 0.7 µg of purified RNA. Gene expression was determined by real-time quantitative PCR (qPCR) using PerfeCTa SYBR Green FasMix ROX (Quanta), with the following set of primers: ACS2; *C. desiccata* (gb. KR150763): forward, 5′-GGGTAATGCGTGCCACAAAG; reverse, 5′-AAAACCACGTGAAAGGCATGA; *D. tertiolecta* (gb. KR150764): forward, 5′-CCTGGGTAAGGTC CGAAGTAG; reverse, 5′- GGGTAGAGCTGCAAGAGGAGT; *cw15* (XM_001700178.1): forward, 5′-TGTGCAGGACAA GCAACAGTA; reverse, 5′-ACTTGGTCTCCCAGTGGAACT; ACL-b expression was determined by the following set of primers: *C. desiccata* (gb. KR150762): forward, 5′-GTCGCACC ACCCCTTCTGT; reverse, 5′-TGAGGGATGGCGACTTCTTC; *D. tertiolecta* (CBZS4528): forward, 5′-CACTCGCTACTTGGA TTACGC; reverse, 5′-AACGTTCATCACCAAGTTTGC: *cw15* (XM_001701903.1) forward, 5′-TACTACGGCGTCAGCATGTC; reverse, 5′-ATGAACTTGGTGGCGTACTTG.

The level of expression was normalized according to selected endogenous genes, as follows: *C. desiccata*: ACTIN (gb. KP293895); forward, 5′-CGCGACATCAAGGAGAAGCT; reverse, 5′-TCTGAAGGGTGGAGGAAGCA; *D. tertiolecta*: 18S (g.b EF473729); forward, 5′-CGCGCTACACTGATGCATTC; reverse, 5′-GACTCGCGCTTACTAGGCAT; cw15: Receptor of activated protein kinase C (CBLP; jgi. 164254); forward, 5′-CTCCATCAAG ATCTGGGACCT; reverse, 5′-TTCTTGCTGGTGATGTTGAACT.

All reactions were performed with 500nM of each primer and 2–10ng of cDNA, per well.

### Antibodies generation

Polyclonal antibodies for ACS2/ACL-b were raised in rabbits as follows: complete amino acid sequences were applied to IEDB Epitope Prediction and Analysis Tool software (http://tools.immuneepitope.org/main), by which appropriate sequences were selected according to their predicted antigenicity. Peptide sequences were chemically synthesized (peptide synthesis service laboratory, WIS), and conjugated to Imject Maleimide-activated Keyhole limphet haemocyanine (mcKLH, Pierce), according to the manufacturers’ instruction. The KLH-conjugated peptides were injected into rabbits while serum was collected periodically and kept at –20 °C. ACS2: *C. desiccata*, (C)YSNDDVGPRES; *D. tertiolecta*/*C. reinhardtii*, (C)QTFKSLPIPITR; ACL-b, (C)SVGVFSPADVDYI.

### Analysis of protein expression by immunoblot assay

Crude protein extracts were generated from pellets of culture samples containing 2×10^6^ (*D. tertiolecta*/*cw15*) or 2×10^7^ (*C. desiccata*) cells and resuspended in 0.1ml of bursting solution (5mM Hepes pH 7.5, 5mM γ-caproic acid, 1mM benzamidine, 1mM PMSF), followed by the addition of 50 μl of SDS sample buffer. Extracts were then loaded on 12% SDS-PAGE acrylamide for western blot assay. Proteins were transferred to nitrocellulose and incubated overnight at 4 °C with PBS, 0.05% Tween, and 10% low-fat milk (T-PBS milk buffer). The blot was incubated for 1h at room temperature with specific antibodies (anti-ACS2, anti-ACLb, or anti-actin) in T-PBS buffer and washed extensively with T-PBS milk buffer. Next, membranes were incubated at room temperature for 1h with anti-rabbit IgG peroxidase-conjugate diluted 1:20 000 in T-PBS and washed in the same buffer. Bound antibodies were detected by ECL detection system (homemade, using γ-caproic acid and luminol of Sigma-Aldrich).

### EM: immune-gold labelling of ACS2 in *C. desiccata*

Samples were prepared according to the Tokuyasu method (Tokuyasu, 1973). Briefly, *C. desiccata* cells were cultured for 24h in N-deprived medium and fixed in 2% glutaraldehyde and 0.1% acroleine in growth medium for 1h. Following that, cells were washed in growth medium and embedded in 10% gelatin in water. The gelatin was hardened at 4 °C, post-fixed overnight with the fixation medium described above, washed in cacodylate buffer and cut into 0.5mm pieces. The embedded cell pieces were then incubated overnight in 2.3M sucrose, frozen in liquid nitrogen, and cut into 80–90nm slices with an EM FC6 cryo-ultramicrotome (Leica Microsystems). After 30min incubation in blocking solution (0.5% gelatin, 0.5% BSA, 0.2% glycine, 0.1% Tween-20 in PBS), slices were incubated for 2h with anti-ACS2 polyclonal rabbit antibodies in blocking solution (1:150 dilution). Slices were washed with PBS containing 0.2% glycine, incubated for 30min in 10nm colloidal-gold-conjugated goat anti-rabbit antibodies (Electron Microscopy Sciences), diluted 1:20 in blocking solution, and washed in PBS and bi-distilled water. Labelled sections were stained with 2% uranyl acetate, embedded in methyl cellulose, and observed in a Tecnai Spirit Transmission Electron Microscope (FEI) operating at 120kV. Images were recorded using an Eagle 2K×2K CCD camera (FE).

### Pulse labelling: the incorporation of ^14^C-labelled acetate into lipids

^14^C-acetate (40–120 nmol, 2–6 μCi) was added to *D. tertiolecta* (10ml, 3×10^6^ cell/ml), *C. desiccata* (30ml, 3×10^7^ cells/ml) and *C. reinhardtii* (10ml, 3×10^6^ cell/ml) during distinct time points. Cultures were grown in N-deficient medium under constant illumination while samples were collected in 12h intervals and washed with fresh medium supplied with 1mM unlabelled (cold) acetate. Cell pellets were then suspended with 4.5ml of chloroform/methanol/water (2:3:2), vigorously mixed and briefly centrifuged to obtain phase separation. The lower phase (lipids) was separated and used for estimating total incorporation into lipids (50 µl). In order to estimate incorporation into TAG and polar lipids, samples were loaded onto TLC plates and separated as described above. TAG and polar lipid bands were cut and extracted with 1.5ml *n*-hexane for 1h, followed by addition of 10ml scintillation solution. The ^14^C radiolabelling was measured using a Tri-carb liquid scintillation counter (PerkinElmer). Specific activities were then calculated in each sample and normalized to chlorophyll contents.

### Endogenous acetate levels

Samples from control and induced (N-deprived) cells were taken periodically, collected by centrifugation and suspended with 0.2ml extraction buffer (5mM Na-HEPES pH 7.5, 5mM γ-aminocaproic acid, 1mM benzamidine, 1mM PMSF) and glass beads (*C. desiccata*, 2.5×10^9^ cells; *D. tertiolecta*/*C. reinhardtii*, 2×10^8^ cells). Samples (30 µl) from each time point were used for acetate determination by NADH/acetate bio-analysis, according to the manufacturer’s instructions (Megazyme).

### Oxygen measurements

Samples from control and induced (N-deprived) cells, corresponding to a chlorophyll content of 2 µg/ml (2ml), were placed in a 3ml sealed oxygen electrode cell (Hansatech). The samples were illuminated with a high red-light laser LH11 (400 µmol m^–2^ s^–1^) and oxygen levels were measured during light/dark cycles (10min each). Measurements were collected and analysed by Oxy-lab software.

## Results

The authors have previously shown that Ac-CoA is accumulated in response to N deprivation and that this increase could be largely attributed to the rapid induction of ptPDH in a TAG-accumulating alga *C. desiccata* (Avidan *et al.*, 2015). Nevertheless, the existence of an additional plastidic Ac-CoA producer known to play a major role in cellular metabolism of yeast, animal, and plant cells—namely ACS2—has led to the testing of the role of this enzyme in green microalgae following N deprivation.

To this end, a comparative approach was used, as previously reported, using three green microalgae species that differ in TAG accumulation capacity: *Chlorella desiccata*, a marine species that rapidly produces high TAG and low starch levels and thus serve as a TAG accumulation model in the authors’ laboratory (Rachutin Zalogin and Pick, 2014*a*); *Dunaliella tertiolecta*, a halotolerant species that accumulates high starch and moderate TAG levels and has been extensively studied (Ben-Amotz *et al.*, 2009; Rismani-Yazdi *et al.*, 2011); and *C. reinhardtii* (*cw15*), which is grown mixotrophically and accumulates moderate levels of starch and TAG, in which the TAG biosynthesis pathway and regulation have been extensively studied (Goodenough *et al.*, 2014). It has already been shown that, not only the extent, but also the rate of TAG accumulation differs dramatically among these species: whereas in *C. desiccata* TAG accumulation reaches about 70% of maximal level within 24h, in *D. tertiolecta* and *C. reinhardtii*, TAG accumulation at 24h of N deprivation is negligible, but it progressively accumulates for up to 7 d (Avidan *et al.*, 2015).

### Expression patterns of ACS2 and ACL following N deprivation

**Fig. 1.**
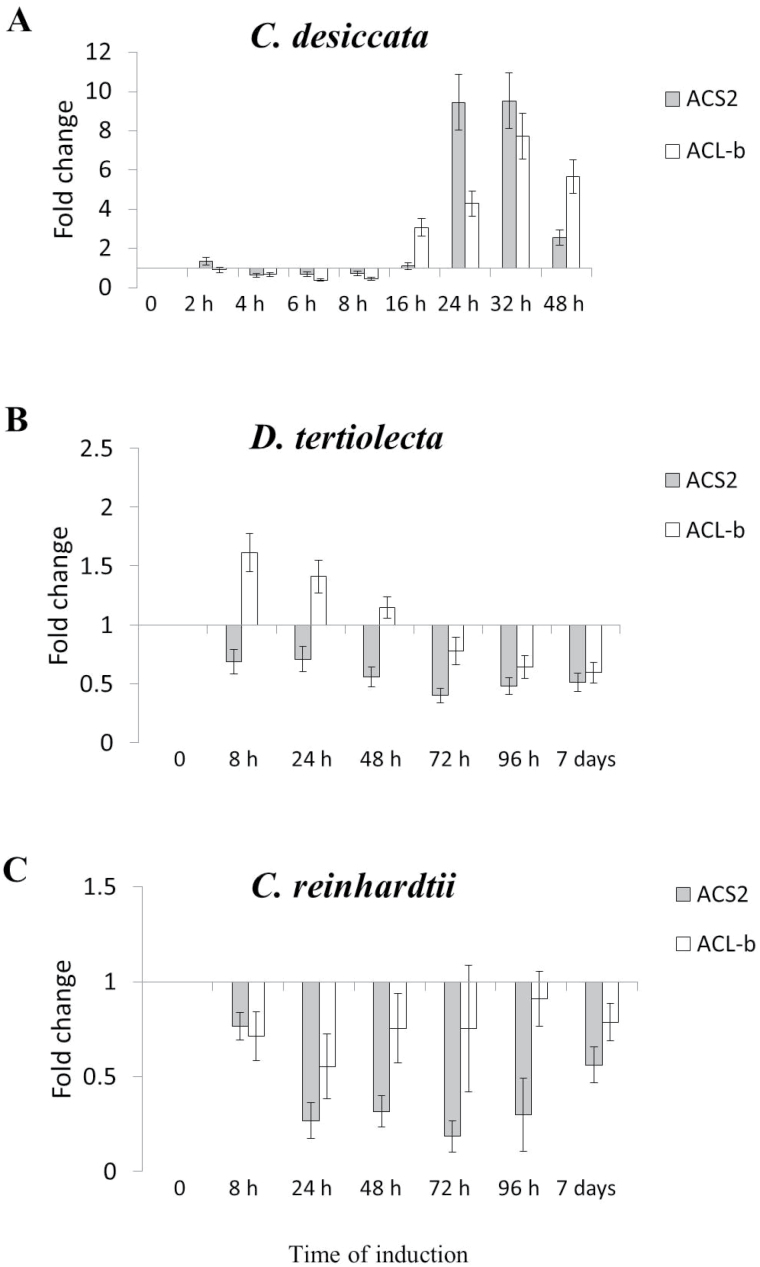
mRNA expression levels of *ACS2* and *ACL-b*. Cultures were grown under continuous high light (400 µmol m^–2^ s^–1^) and induced by N deprivation. Samples taken at the indicated time points were processed for RNA purification and cDNA synthesis using oligo-dt primers. qPCR measurements were normalized to selected endogenous genes and presented as fold-change relative to non-induced cells. (A) *C. desiccata*; (B) *D. tertiolecta*; (C) *C. reinhardtii* (*cw15*) (means ± SD of five independent experiments).

**Fig. 2.**
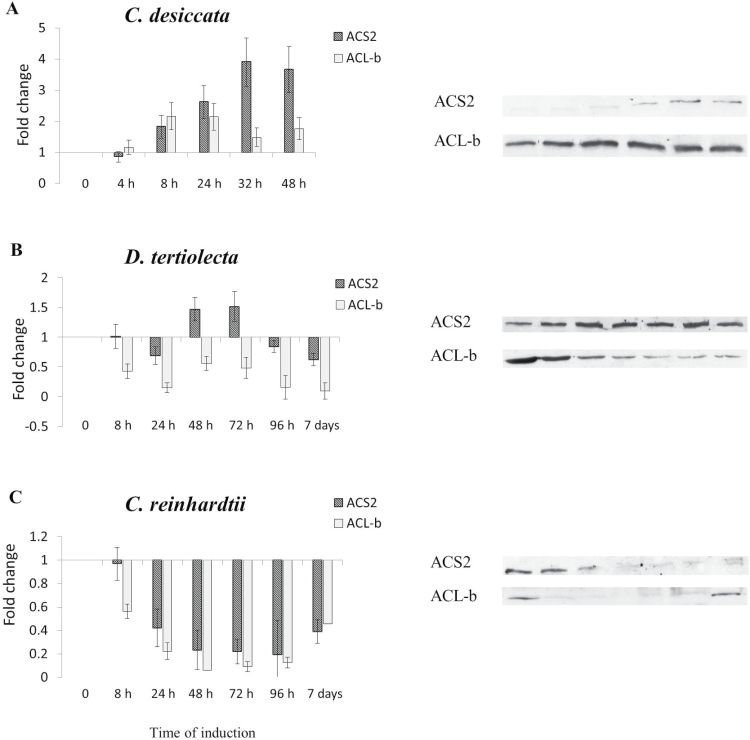
Variations in the expression level of ACS2 and ACL-b proteins following N deprivation. Equal amounts of protein extracts were loaded onto poly-acrylamide gel followed by western blot analysis, using specific anti-ACS2 and anti-ACLb polyclonal antibodies. Expression levels were normalized to actin protein (endogenous gene) in each species. Data are presented as fold-change relative to non-induced cells and quantified by Image-Quant TL program. (A) *C. desiccata*; (B) *D. tertiolecta*; (C) *C. reinhardtii*. (means ± SD of three independent experiments).

As a first step, the transcription pattern of ACS2 in response to N limitations was followed. Considering the prominent role of ACL in FA biosynthesis of non-photosynthetic eukaryotes and prokaryotes (Wellen *et al.*, 2009), it was decided to analyse its expression pattern as well. As depicted in Fig. 1, the transcriptions of these genes vary remarkably among the species. In *C. desiccata*, the transcript level of both genes is upregulated between 16 and 48h of N deprivation. ACS2 transcript levels are elevated by 10-fold reaching a maximum at 24–32h, whereas ACL-b peak expression (eight-fold) occurs at 32h. Conversely, none of the genes was significantly upregulated in *cw15* or in *D. tertiolecta*. Similarly, an immunoblot analysis of protein abundance (Fig. 2) confirms that both genes are significantly upregulated only in *C. desiccata*. The upregulation in *C. desiccata* is surprising since previous experiments in plants have demonstrated that expression of ACL and ACS mRNA levels do not share spatial or temporal correlation with FA and TAG synthesis (Bao *et al.*, 2000; Ke *et al.*, 2000). This raises questions regarding their possible involvement in carbon metabolism and TAG synthesis in *C. desiccata*.

### Chloroplastic localization of ACS2

**Fig. 3.**
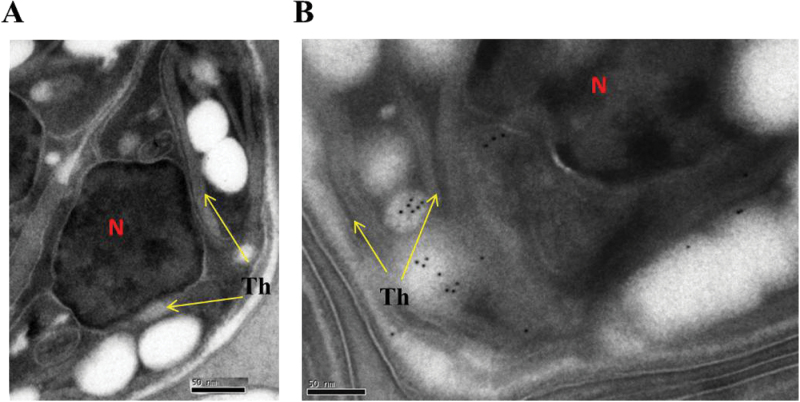
Immunogold labelling of ACS2 confirms chloroplastic localization in *C. desiccata*. Non-induced (A) and 24h induced (B) cells were subjected to immunogold labelling using anti-ACS2 polyclonal antibodies as described in *Materials and methods*. Chloroplastic protein clusters are detectable in induced samples only (black dots). N, nucleus; Th, thylakoid membrane.

In order to validate ACS2 localization, *C. desiccata* cells were induced by N deprivation for 24h and prepared for immune-gold labelling by EM, using polyclonal ACS2 antibodies generated in the lab (described in *Materials and methods*). As visualized in Fig. 3A, 3B, gold particles are clustered within the chloroplast boundary of induced cells only. Interestingly, clusters of particles were observed on surfaces of structures identified as plastidic lipid droplets (Davidi *et al.*, 2014). Such observation validates the chloroplastic localization of ACS2, consistent with a chloroplast signal-peptide sequence found in its 5′-end, as previously described for this gene (Ke *et al.*, 2000).

### Is ACS2 expressed as part of the PDH-bypass?

Several studies in plants have associated ACS2 with the PDH-bypass (Mellema *et al.*, 2002; Lin and Oliver, 2008). To find out whether the upregulation of ACS2 in *C. desiccata* under nitrogen deprivation is also part of the activation of the PDH-bypass, several parameters known to be associated with this pathway were tested in each of the three algal strains.

#### ^14^C-Acetate incorporation into lipids

**Fig. 4.**
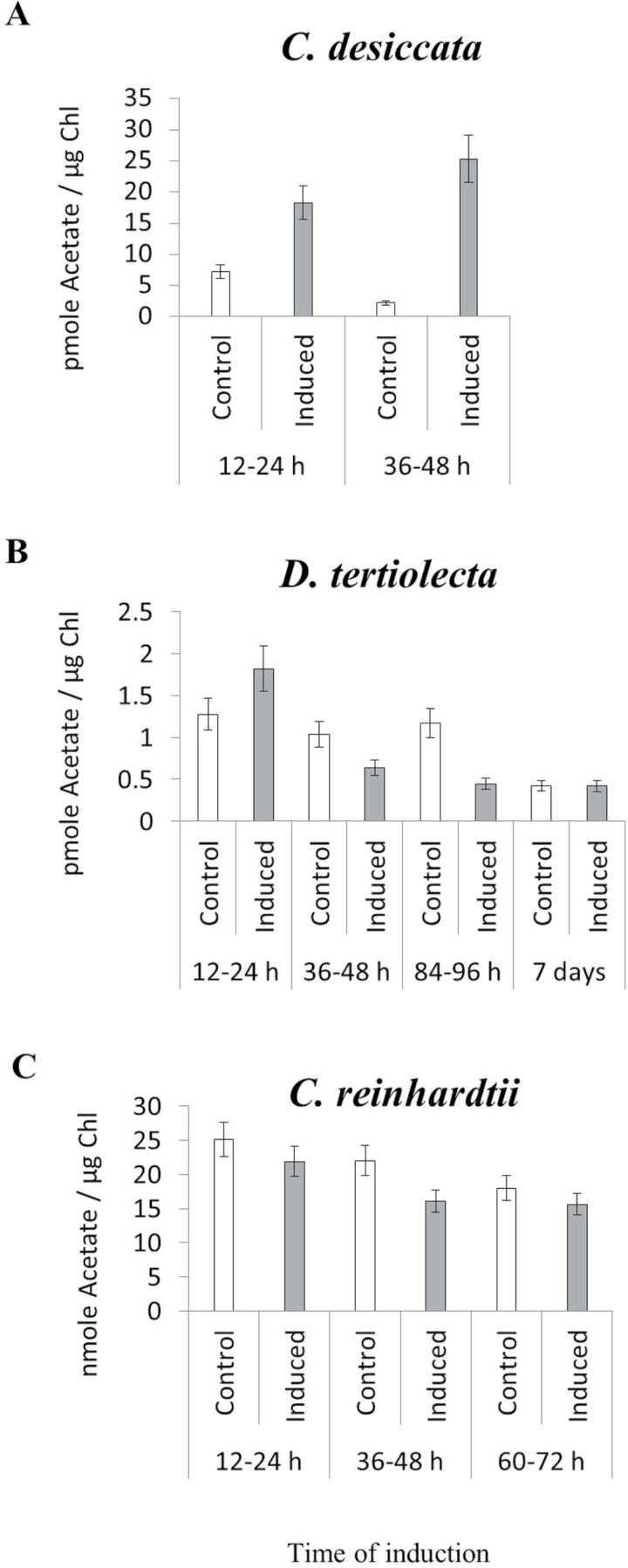
Incorporation of ^14^C-acetate into lipids at different stages during N deprivation. Exogenous ^14^C-acetate was added to N-deprived *C. desiccata* (A), *D. tertiolecta* (B), and *C. reinhardtii* (C) cultures at the indicated time windows during N deprivation and to control cultures. The rate of incorporation into lipids was used as a measure for ACS2 protein activity. (means ± SD of three independent experiments).

Previous experiments have shown a correlation between ACS protein levels and the incorporation of ^14^C-acetate into FA in spinach and oilseed rape (Roughan, 1994; Roughan and Ohlrogge, 1996), and as such, it may serve as a parameter for *in vivo* ACS activity. Accordingly, whether the rate of ^14^C-labelled acetate incorporation into lipids correlates with upregulation of ACS2 was tested (Fig. 4). In order to distinguish between baseline and induced incorporation, labelled acetate was added at several specific time windows and its incorporation into lipids analysed during 12h intervals. Rates of acetate incorporation into lipids were expressed per microgram of chlorophyll (which is the best parameter for comparison in view of the different sizes and chlorophyll content of the three species) and corrected for the drop in chlorophyll/cell during N deprivation. As shown in Fig. 4, the extents of incorporation into lipids in response to N starvation vary greatly among the three strains: only in the high TAG accumulator *C. desiccata,* was acetate incorporation significantly increased in response to N deprivation, particularly at the 36–48h time window, consistent with maximal upregulation of ACS2 during that time in this species. In *D. tertiolecta*, there was a small increase at 12–24h, followed by a reduced incorporation at later time intervals. In *cw15*, high incorporation into lipids was observed, but it was not elevated under N deprivation (Fig. 4C). High acetate incorporation rate is expected, since *cw15* is normally grown with acetate as its major carbon source. Taken together, only in *C. desiccata* was a correlation between peak incorporation (36–48h) and ACS2 expression (32h) observed, suggesting that ACS2 may indeed contribute to acetate incorporation into TAG at the late stages of N deprivation in this species.

#### Endogenous acetate production

**Fig. 5.**
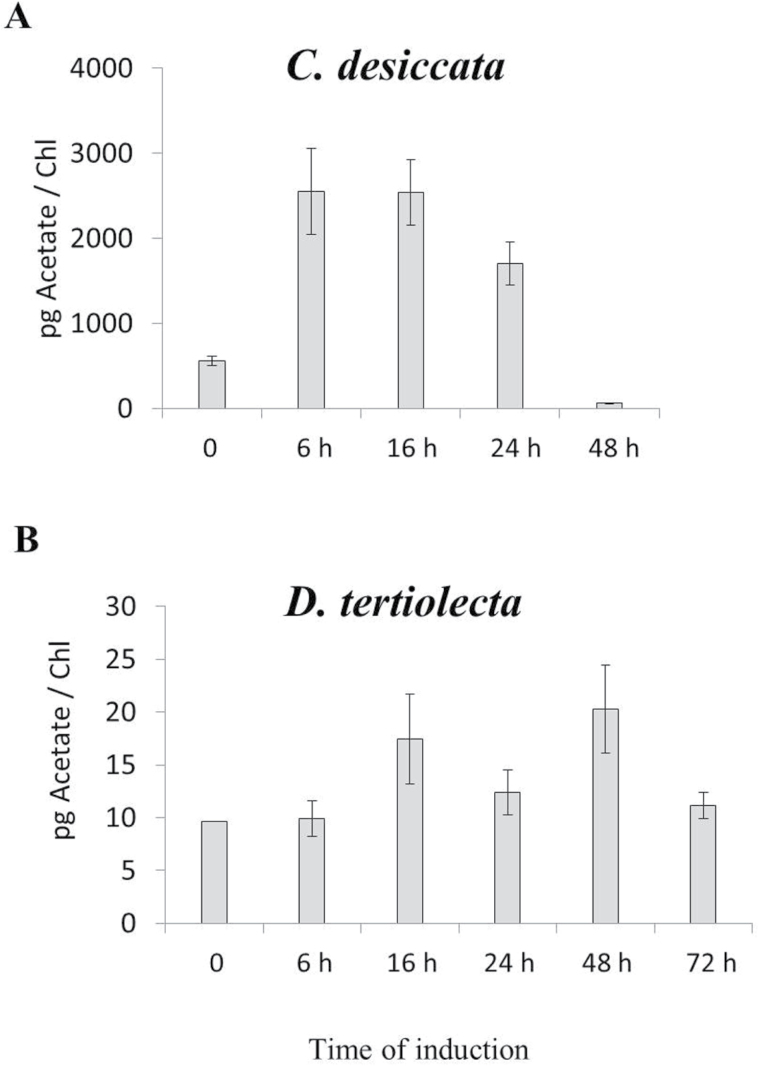
Endogenous acetate production following N deprivation. Samples from control and induced cultures of *C. desiccata* (A) and *D. tertiolecta* (B) were collected and quenched. Cell extracts were analysed for acetate levels by the NADH/acetate bioassay. Acetate levels are expressed per microgram of chlorophyll and corrected for the drop in Chl/cell as in Fig. 4 (means ± SD of three independent experiments).

Following the induction of the PDH-bypass, pyruvate is converted to acetaldehyde, which in turn is converted to ethanol. Since the ability of cells to endure increasing concentrations of acetaldehyde and ethanol is limited, acetaldehyde must be detoxified into acetate by ACS. The activation of ACS2 during N starvation in *C. desiccata* implies that acetate is indeed formed within this timeframe. In order to detect endogenous production of acetate, which could provide further indication for the activation of the PDH-bypass, an assay was used that is composed of coupled enzymatic reactions which lead to the production of Ac-CoA and NADH, proportional to acetate levels in a given extract (described in *Materials and methods*). *C. reinhardtii cw15* was excluded from this analysis because it is cultured in an acetate-containing medium. Control and induced cells of *C. desiccata* and of *D. tertiolecta* were deprived of nitrogen, collected at various time points, quenched, and lysed for NADH/acetate bio-analysis. As shown in Fig. 5, the amount of acetate produced by *C. desiccata* is 100-fold higher than that in *D. tertiolecta*. The rise in endogenous acetate level is transient, reaching a maximum after 6h and decreasing thereafter, in inverse correlation with the induction of ACS2 (at 8–32h, Fig. 2A), consistent with the early induction of the PDH-bypass in *C. desiccata* and not in *D. tertiolecta*.

#### Expression of alcohol dehydrogenase

**Fig. 6.**
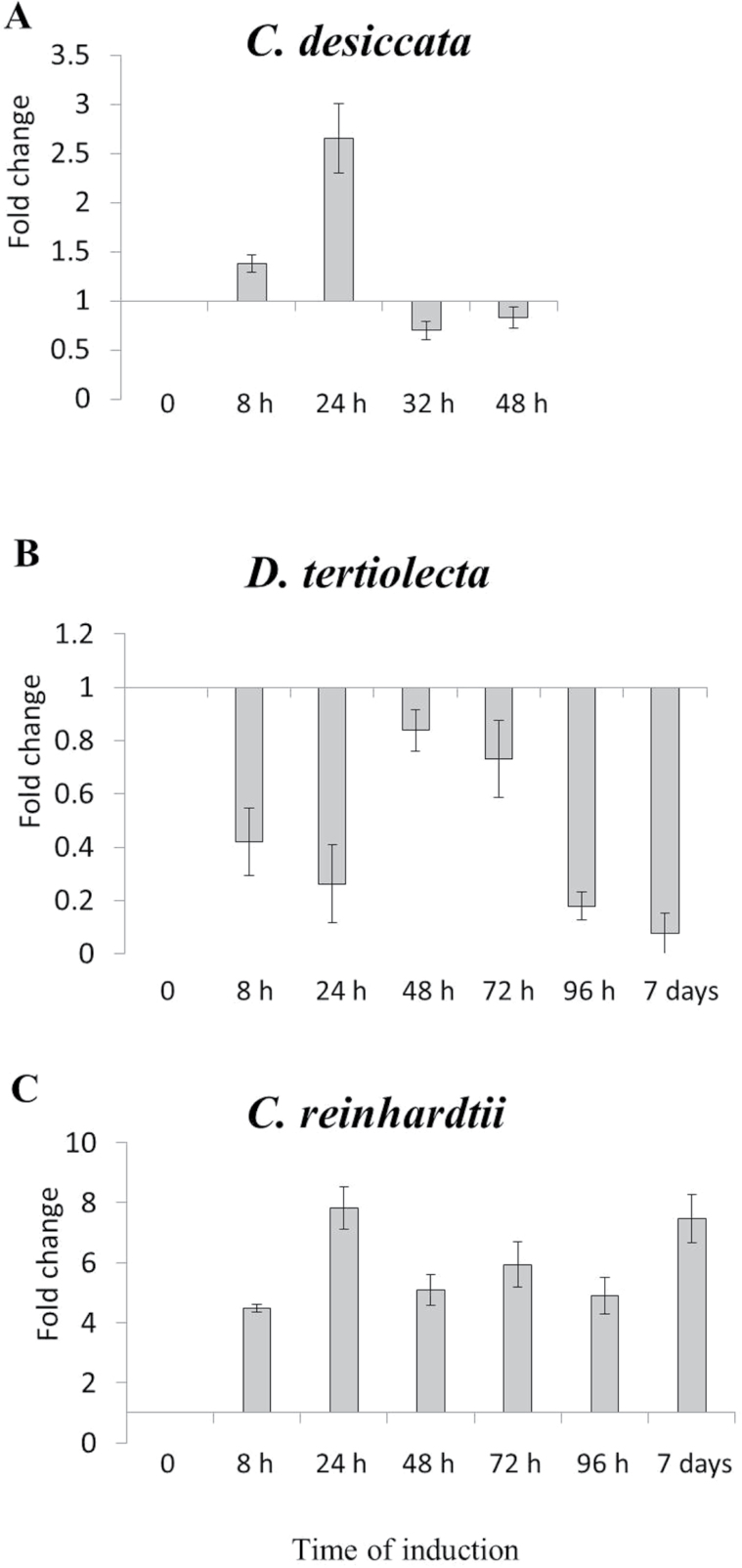
The expression of ADH1 protein following N deprivation. Crude protein extracts were analysed for ADH1 expression, as described in Fig. 2, using anti-*E. coli* adhE antibodies (Hemschemeier *et al.*, 2008) (means ± SD of three independent experiments).

As a major component of the PDH-bypass (Zabalza *et al.*, 2009), localized upstream to ACS, the activity of alcohol dehydrogenase (ADH)1 is expected to be induced at the early stages of activation of the bypass. Indeed, an immunoblot analysis with well-characterized polyclonal antibodies (Hemschemeier *et al.*, 2008) revealed that ADH1 is rapidly and transiently upregulated in *C. desiccata* (8–24h) while gradually downregulated in *D. tertiolecta* in response to N starvation (Fig. 6A, 6B). Moreover, the peak expression after 24h precedes that of ACS2 (32h) in *C. desiccata*, consistent with activation of the pathway in this species and not in Dunaliella. Additionally, as this pathway was suggested previously to play a role in detoxification of fermentation products (e.g. ethanol and acetaldehyde), the transient sharp expression in *C. desiccata* may imply that these intermediates are rapidly and transiently accumulated during that time. In *C. reinhardtii*, ADH1 refers to the dual function alcohol/acetaldehyde dehydrogenase enzyme and is continuously overexpressed to high levels following N deprivation. Considering that ACS2 was downregulated under these conditions, the upregulation of ADH1 is most probably linked to various bacterial-like fermentation pathways known to be induced in this species following N starvation, rather than to the PDH-bypass (Gibbs *et al.*, 1986; Oa and Bo, 2005; Terashima *et al.*, 2010).

#### Respiration and oxygen evolution

Nitrogen assimilation in plants and algae is mainly mediated by plastidic nitrate reductase and glutamine oxoglutarate amidotransferase, and depends on a constant supply of carbon, amino acids (Glu, Gln), and reducing equivalents from mitochondria, cytoplasm, and plastids (e.g, malate, 2-oxoglutarate) (Linka and Weber, 2005; Rodolfi *et al.*, 2009; Nunes-Nesi *et al.*, 2010; Blaby *et al.*, 2013; Schmollinger *et al.*, 2014). Accordingly, the metabolism of carbon and of nitrogen are tightly linked, hence, any decrease in N availability may affect cellular C/N ratio. Since TAG accumulation in algae is achieved through N limitations, the rate of carbon exchange between chloroplast and mitochondria is significantly hampered, leading to increased accumulation of ATP and reducing equivalents (NADPH) (Hoefnagel *et al.*, 1998; Padmasree *et al.*, 2002; Raghavendra and Padmasree, 2003). Considering that cells do not divide under these conditions, constant pyruvate supply may overload and limit the activity of mtPDH and thus also the tricarboxylic acid and mitochondrial respiration. As a regulator of pyruvate and oxygen levels, the induction of the PDH-bypass would then tunnel a portion of the pyruvate towards ethanol production, as part of an aerobic fermentation. Consequently, it should reduce the amount of pyruvate respired, which in turn, would sustain higher mitochondrial activity, as previously proposed (Zabalza *et al.*, 2009).

**Fig. 7.**
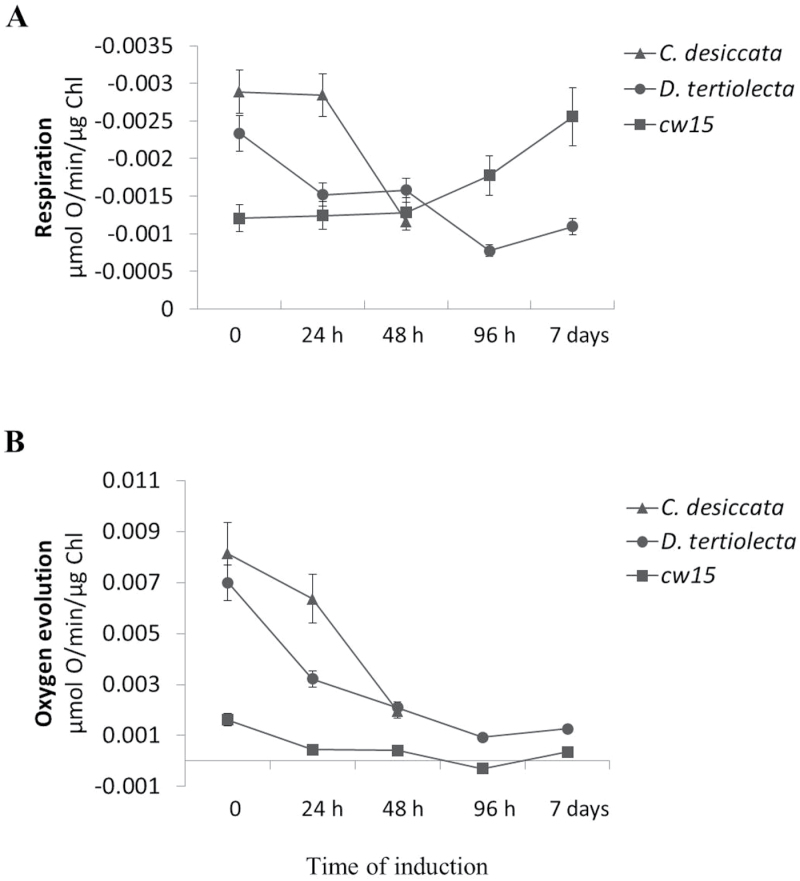
The effect of N deprivation on respiration and on photosynthesis. Cell samples taken at the indicated time points of induction were incubated in an oxygen electrode cell and exposed to cycles of red-light illumination (400 µmol m^–2^ s^–1^) and dark periods as described in *Materials and methods*. Oxygen evolution represents the difference between oxygen levels in light and dark periods. (A) oxygen uptake (respiration); (B) oxygen evolution (photosynthesis) (means ± SD of three independent experiments).

To test whether this is the case also in the microalgae, variations in oxygen production (photosynthesis) and uptake (respiration) of light and dark-adapted control and N-deprived cells were measured (Fig. 7). Significant changes in the response to N deprivation were observed among the three species. In *D. tertiolecta* and in *C. desiccata*, oxygen evolution and respiration rates changed in a similar manner in response to N deprivation, but with one difference: at the first 24h of N deprivation, *D. tertiolecta* exhibited a sharp decrease in both respiration and photosynthesis, whereas in *C. desiccata*, there was only a small (photosynthesis) or no (respiration) detectable decrease. After 48h, both species exhibited a sharp decrease in photosynthesis and respiration. In *C. reinhardtii*, both basal respiration and photosynthesis rates in control cells were much lower (by two- to five-fold). Following induction, net oxygen production dropped steeply, whereas respiration rate rose over time (two-fold), probably reflecting degradation of chloroplasts for buildup of mitochondrial components, consistent with previous reports on adaptation of heterotrophic algae to N deprivation (Miller *et al.*, 2010; Schmollinger *et al.*, 2014).

Taken together, the 24h delay in the inhibition of respiration and photosynthesis observed in N-deprived *C. desiccata*, and the indications for the activation of the PDH-bypass during this time (ACS2, ADH1, enhanced acetate production and incorporation), suggest that the PDH-bypass may be involved in protecting mitochondrial and chloroplast metabolism to enable high TAG accumulation activity under N deprivation in *C. desiccata*.

## Discussion

It has been estimated that in rapidly growing plant tissues, such as pollen tubes and roots, 20–50% of glycolytic glucose is fermented through the PDH-bypass (Bucher *et al.*, 1995; Tadege and Kuhlemeier, 1997; Mellema *et al.*, 2002). Therefore, it is interesting to assess whether the primary role of the pathway in *C. desiccata* is to supply Ac-CoA for FA biosynthesis or alternatively, to support respiration and photosynthesis. In order to roughly estimate how significant is the flux through this pathway towards FA biosynthesis in *C. desiccata*, the levels of incorporation of acetate and bicarbonate into TAG were compared. Calculated levels of ^14^C-acetate and ^14^C-bicarbonate incorporation in *C. desiccata* give values of approximately 10ng/µg Chl and 10 µg/µg Chl for acetate and bicarbonate, respectively (Supplementary Fig. S1, available at *JXB* online). These calculations, however, should be considered with caution. The low calculated acetate incorporation rate may be underestimated because it is not known how much ^14^C-acetate penetrates the chloroplast and to what extent it is ‘diluted’ with endogenous acetate. Nevertheless, although no strict flux-related determinations can be drawn from these calculations, the three orders of magnitude difference between these values suggests that the contribution of ACS2 to the overall carbon tunnelling towards lipids in this alga is most probably negligible. These findings are in agreement with previous reports in *Arabidopsis* leaves (Lin and Oliver, 2008), which implied that the role of ACS2 is to remove its substrate—acetate, and its toxic precursors—ethanol and acetaldehyde, rather than supplying Ac-CoA for lipid biosynthesis. In contrast, in heterotrophically grown *C. reinhardtii*, that were induced to accumulate TAG by an acetate boost, ACS2 and ACS3 seem to serve as a major carbon assimilation pathway for TAG biosynthesis (Goodenough *et al.*, 2014).

Several lines of evidence suggest that ACS2 is induced as part of the PDH-bypass in *C. desiccata* under N deprivation: of the three major enzymes comprising the PDH-bypass—pyruvate decarboxylase (PDC), ACS, and ADH—at least two are upregulated in *C. desiccata*. Unfortunately, PDC mRNA could not be detected, possibly because the enzyme in this species may differ in sequence from other green algae. Also, the enhanced production of endogenous acetate and the incorporation of acetate into lipids in this alga are consistent with activation of the PDH-bypass. Moreover, the finding that, endogenous acetate peaks at 6–16h of N deprivation and decreases thereafter between 16 and 48h, correlated with the maximal induction of ACS2 at 24–32h, suggests that ACS2 is a major consumer of acetate. However, the role of the PDH-bypass in this alga is not clear.

Upregulation of the PDH-bypass under nitrogen deprivation was recently reported also in another oleaginous alga *Nannochloropsis* (Li *et al.*, 2014), indicating that this metabolic pathway may be linked to the high energy demands needed for massive TAG biosynthesis under N deprivation.

The PDH-bypass may have diverse roles in different organisms and even in different tissues in the same organism. In yeast, it is induced under conditions where the glycolytic activity exceeds the respiratory capacity, such as at high glucose or at limiting oxygen. It has been proposed that the limiting factor under these conditions is the turnover of mtPDH, and the bypass serves to divert part of the excess pyruvate from reaching the mitochondria and thus maintain the energetic status (Holzer and Goedde, 1957; Berg and Steensma, 1995; Pronk *et al.*, 1996, Otterstedt *et al.*, 2004). In plants, the proposed roles of the PDH-bypass are to relieve mitochondrial over-energization, to enable cells to maintain respiratory energetic and metabolic activities (Shiao *et al.*, 2002; Boamfa *et al.*, 2005; Gass *et al.*, 2005; Zabalza *et al.*, 2009), and/or in detoxification of fermentation products (Ke *et al.*, 2000; Lin and Oliver, 2008). In pollen tubes of plants, activation of the bypass supports respiration and lipid biosynthesis (Mellema *et al.*, 2002), and it has been proposed that its role is to relieve the limited capacity of PDH to metabolize pyruvate (Gass *et al.*, 2005). The role of the PDH-bypass in *C. desiccata* is not clear.

One possibility is that similar to yeast and plants, activation of the PDH-bypass serves to protect respiratory and/or photosynthetic activity under N deprivation. The delayed inhibition of respiratory and photosynthetic activities in *C. desiccata* in comparison to *D. tertiolecta* is consistent with this possibility. It is also in line with a previous finding that this species is relatively resistant to sodium azide in comparison to other green algae: sodium azide induces massive accumulation of TAG in *C. desiccata* with minimal growth retardation through inhibition of nitrate reductase, which mimics nitrogen deprivation (Rachutin-Zalogin and Pick, 2014*a*, 2014*b*). It is likely that the reason for the resistance of *C. desiccata* to growth retardation under these conditions results from activation of the PDH-bypass.

Is it possible that activation of the PDH-bypass serves to protect PDH from overflow of pyruvate, as proposed in yeast and in plants? The answer to this question is complicated because in algae, as in plants, there are two distinct PDH enzymes, one in the mitochondrion (mtPDH), serving to generate Ac-CoA for mitochondrial respiration, and one in the plastids (ptPDH), serving to generate Ac-CoA mainly for FA biosynthesis. Both possibilities are equally intriguing.

The possibility of protecting mtPDH is attractive for several reasons: first, it is consistent with the delayed inhibition of mitochondrial respiration, as discussed above. Second, it has been shown that under nitrogen limitation in green algae, protein synthesis and chlorophyll contents are dramatically reduced, and the bypass may be important for sustaining efficient mitochondrial activity, and exchange of metabolites and reducing equivalents between the mitochondria and chloroplast, which are needed for the high metabolic demands during massive TAG biosynthesis (Terashima *et al.*, 2010; Banti *et al.*, 2013; Schmollinger *et al.*, 2014). A recent study has shown that the mitochondrial TCA cycle in Nannochloropsis is upregulated under nitrogen deprivation (Li *et al.*, 2014), consistent with this idea.

Third, it is consistent with the activation of ACL, whose significance is not yet clear. As noted above, this cytoplasmatic enzyme was shown to serve as the major supplier of Ac-CoA for FA biosynthesis in non-photosynthetic organisms (Wellen *et al.*, 2009). This raises an interesting possibility that activation of FA biosynthesis in the cytoplasm may contribute to the massive TAG biosynthesis under N deprivation in this oleaginous green alga. In preliminary studies, there were two indications that support this possibility: treatment of N-deprived *C. desiccata* with 100 μM sethoxydim—a selective inhibitor of the homomeric Ac-CoA carboxylase, which specifically inhibits synthesis of FA in the cytoplasm in dicotyledonous plants (Slabas *et al.*, 2002; Sasaki and Nagano, 2004; Belkebir *et al.*, 2006)—inhibited TAG accumulation by about 50%, whereas 5mM 1,2,3-benzenetricarboxylate—a specific inhibitor of the mitochondrial citrate transporter (Aluvila *et al.*, 2010), which is expected to decrease the availability of cytoplasmic citrate required for Ac-CoA production by ACL—inhibited TAG accumulation by about 30%. These inhibitors did not inhibit TAG accumulation in N-deprived *D. tertiolecta* or in *C. reinhardtii* (Supplementary Fig. S1, available at *JXB* online). Further studies are needed to validate the contribution of cytoplasmic Ac-CoA production by ACL to TAG accumulation in oleaginous algae. However, if true, the activity of ACL, which converts citrate to Ac-CoA, would require enhanced efflux of citrate from mitochondria to the cytoplasm, which would drain the TCA cycle and necessitate constant replenishment of TCA intermediates and increased supply of pyruvate to mtPDH. Thus, activation of the PDH-bypass may be important to control mtPDH due to its expected enhanced activity under these conditions.

**Fig. 8.**
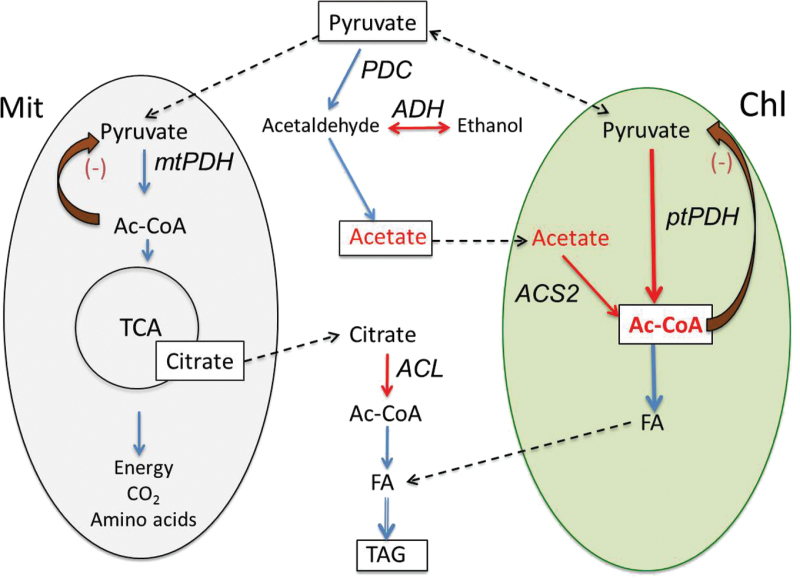
Proposed scheme of interactions between Ac-CoA producers in *C. desiccata*. Depicted is a proposed mechanism by which the PDH-bypass controls the availability of pyruvate to the mitochondrial (Mit, grey) mtPDH and to the chloroplastic (Chl, green) ptPDH under N deprivation. Solid lines, enzymatic reactions; broken lines, diffusion of transport; red lines, induced enzymes or substrates; curved solid brown arrows, product inhibition; italics, enzyme names.

The possibility that the bypass is activated to protect ptPDH is equally intriguing: ptPDH is sensitive to product inhibition by NADH/NADPH and by Ac-CoA (reviewed in Tovar-Mendez *et al.*, 2003). As mentioned above, it was found that during N deprivation there is a dramatic rise in Ac-CoA levels in the chloroplast of *C. desiccata* (Avidan *et al.*, 2015), which may limit ptPDH activity. Furthermore, there was upregulation of ptPDH under nitrogen deprivation, suggesting that the activity of this enzyme is limiting under these conditions. Notably, the large increase in Ac-CoA and activation of ptPDH were observed only in the oleaginous *C. desiccata*, in which the bypass is upregulated, but not in other green algae species that do not accumulate high levels of TAG. Thus, it is proposed that the PDH-bypass in this alga may be activated in order to control and/or protect ptPDH to enable rapid production of Ac-CoA within the chloroplast that is utilized for massive FA and TAG biosynthesis. A schematic representation of the proposed enzymatic reactions involved in Ac-CoA production in *C. desiccata* under N deprivation is depicted in Fig. 8. According to the proposed scheme, part of the pyruvate that is produced in the chloroplast that exceeds the capacity of ptPDH to produce Ac-CoA, is transported to the cytoplasm, where it may have two fates: it can be either be processed by the PDH-bypass, converted to acetate—which may diffuse back into the chloroplast to be converted to Ac-CoA by ACS2, or it may enter the mitochondria, be converted to Ac-CoA by mtPDH, and incorporated into the TCA cycle. Mitochondrial pyruvate may also serve to replenish the level of TCA intermediates that is constantly depleted, partially by efflux of citrate to the cytoplasm, where it is converted to Ac-CoA by ACL. The produced cytoplasmic Ac-CoA may be utilized to produce FA in the cytoplasm, increasing the FA pool needed for massive TAG biosynthesis.

In summary, activation of the PDH-bypass in *C. desiccata* is correlated with enhanced TAG biosynthesis in this species. The estimated low carbon assimilation from acetate into TAG suggests that its primary role is not to provide Ac-CoA for TAG biosynthesis. It is proposed that the main role of the PDH-bypass during N starvation is to maintain mtPDH activity and to control the level of pyruvate reaching ptPDH in order to optimize Ac-CoA production in the chloroplast.

## Supplementary data

Supplementary data are available at *JXB* online.

Fig. S1. Effects of citrate efflux and hmACCase inhibitors on TAG level.

Supplementary Data

## Abbreviations:

Ac-CoA: acetyl-coenzyme A
ACL: ATP citrate lyase
ACS: Ac-CoA synthase
ADH: alcohol dehydrogenase
FA: fatty acids
ptPDH: plastidic pyruvate dehydrogenase
TAG: triacylglycerol.

## References

[CIT0001] Aluvila S Sun J Harrison DHT Walters DE Kaplan RS 2010 Inhibitors of the mitochondrial citrate transport protein : Validation of the role of substrate-binding residues and discovery of the first purely competitive inhibitor. Molecular Pharmacology . 77, 26–34. 19843634 10.1124/mol.109.058750 PMC2802432

[CIT0002] Avidan O Brandis A Rogachev I Pick U 2015 Enhanced acetyl-CoA production is associated with increased triglyceride accumulation in the green alga *Chlorella desiccata* . Journal of Experimental Botany (in press). 10.1093/jxb/erv166 PMC4473976 25922486

[CIT0003] Banti V Giuntoli B Gonzali S 2013 Low oxygen response mechanisms in green organisms. International Journal of Molecular Sciences 14, 4734–4761. 23446868 10.3390/ijms14034734 PMC3634410

[CIT0004] Bao X Focke M Pollard M Ohlrogge J 2000 Understanding *in vivo* carbon precursor supply for fatty acid synthesis in leaf tissue. Plant Journal . 22, 39–50. 10792819 10.1046/j.1365-313x.2000.00712.x

[CIT0005] Beisson F Koo A Ruuska S 2003 Arabidopsis genes involved in acyl lipid metabolism. A 2003 census of the candidates, a study of the distribution of expressed sequence tags in organs, and a web-based database. Plant Physiology 132, 681–697. 12805597 10.1104/pp.103.022988 PMC167007

[CIT0006] Belkebir A De Paepe R Trémolières A Aïd F Benhassaine-Kesri G 2006 Sethoxydim affects lipid synthesis and acetyl-CoA carboxylase activity in soybean. Journal of Experimental Botany 57, 3553–3562. 16968879 10.1093/jxb/erl108

[CIT0007] Ben-Amotz A Polle JEW Subba Rao DV 2009 The alga Dunaliella : biodiversity, physiology, genomics and biotechnology ( B-AA. PJEW S Rao, Ed.). Enfield.

[CIT0008] Berg MA Steensma HY 1995 ACS2, a *Saccharomyces cerevisiae* gene encoding acetyl-coenzyme A synthetase, essential for growth on glucose. European Journal of Biochemistry 231, 704–713. 7649171 10.1111/j.1432-1033.1995.tb20751.x

[CIT0009] Blaby IK Glaesener AG Mettler T 2013 Systems-level analysis of nitrogen starvation-induced modifications of carbon metabolism in a *Chlamydomonas reinhardtii* starchless mutant. Plant Cell 25, 4305–4323. 24280389 10.1105/tpc.113.117580 PMC3875720

[CIT0010] Boamfa EI Veres a H Ram PC Jackson MB Reuss J Harren FJM 2005 Kinetics of ethanol and acetaldehyde release suggest a role for acetaldehyde production in tolerance of rice seedlings to micro-aerobic conditions. Annals of Botany 96, 727–736. 16093270 10.1093/aob/mci224 PMC4247039

[CIT0011] Boulton C Ratledge C 1981 Correlation of lipid accumulation in yeasts with possession of ATP: citrate lyase. Journal of General Microbiology 127, 169–176.

[CIT0012] Boyle NR Page MD Liu B 2012 Three acyltransferases and nitrogen-responsive regulator are implicated in nitrogen starvation-induced triacylglycerol accumulation in Chlamydomonas. Journal of Biological Chemistry 287, 15811–15825. 22403401 10.1074/jbc.M111.334052 PMC3346115

[CIT0013] Bucher M Brander KA Sbicego S Mandel T Kuhlemeier C 1995 Aerobic fermentation in tobacco pollen. Plant Molecular Biology 28, 739–750. 7647304 10.1007/BF00021197

[CIT0014] Davidi L Shimoni E Khozin-Goldberg I Zamir A Pick U 2014 Origin of β-carotene-rich plastoglobuli in *Dunaliella bardawil* . Plant Physiology 164, 2139–2156. 24567188 10.1104/pp.113.235119 PMC3982768

[CIT0015] Dean AP Sigee DC Estrada B Pittman JK 2010 Using FTIR spectroscopy for rapid determination of lipid accumulation in response to nitrogen limitation in freshwater microalgae. Bioresource Technology 101, 4499–4507. 20153176 10.1016/j.biortech.2010.01.065

[CIT0016] Fan J Yan C Andre C Shanklin J Schwender J Xu C 2012 Oil accumulation is controlled by carbon precursor supply for fatty acid synthesis in *Chlamydomonas reinhardtii* . Plant and Cell Physiology 53, 1380–1390. 22642988 10.1093/pcp/pcs082

[CIT0017] Fatland BL Ke J Anderson MD Mentzen WI Cui LW Allred CC Johnston JL Nikolau BJ Wurtele ES 2002 Molecular characterization of a heteromeric ATP-citrate lyase that generates cytosolic acetyl-coenzyme A in Arabidopsis. Plant Physiology 130, 740–756. 12376641 10.1104/pp.008110 PMC166603

[CIT0018] Fatland BL Nikolau BJ Wurtele ES 2005 Reverse genetic characterization of cytosolic acetyl-coA generation by ATP-citrate lyase in Arabidopsis. American Society of Plant Biolgists 17, 182–203. 10.1105/tpc.104.026211 PMC544498 15608338

[CIT0019] Gass N Glagotskaia T Mellema S Stuurman J Barone M Mandel T Roessner-tunali U Kuhlemeier C 2005 Pyruvate decarboxylase provides growing pollen tubes with a competitive advantage in Petunia. Plant Cell 17, 2355–2368. 15994907 10.1105/tpc.105.033290 PMC1182494

[CIT0020] Gibbs M Gfeller RP Chen C 1986 Fermentative metabolism of *Chlamydomonas reinhardtii* . Plant Physiology 2, 160–166. 16664985 10.1104/pp.82.1.160 PMC1056083

[CIT0021] Goodenough U Blaby I Casero D 2014 The path to triacylglyceride obesity in the *sta6* strain of *Chlamydomonas reinhardtii* . Eukaryotic Cell 13, 591–613. 24585881 10.1128/EC.00013-14 PMC4060482

[CIT0022] Goodson C Roth R Wang ZT Goodenough U 2011 Structural correlates of cytoplasmic and chloroplast lipid body synthesis in *Chlamydomonas reinhardtii* and stimulation of lipid body production with acetate boost. Eukaryotic Cell 10, 1592–1606. 22037181 10.1128/EC.05242-11 PMC3232719

[CIT0023] Gorman DS Levine RP 1965 Cytochrome f and plastocyanine: their sequence in the electron transport chain of *Chlamydomonas reinhardtii* . Proceedings of the Natlional Academy of Science USA 54, 1665–1669. 10.1073/pnas.54.6.1665 PMC300531 4379719

[CIT0024] Guillard R Ryther J 1962 Studies of marine planktonic diatoms: I. *Cyclotella nana* Hustedt, and *Detonula confervacea* (Cleve) Gran. Canadian Journal of Microbiology 8, 229–239. 13902807 10.1139/m62-029

[CIT0025] Hemschemeier A Jacobs J Happe T 2008 Biochemical and physiological characterization of the pyruvate formate-lyase Pfl1 of *Chlamydomonas reinhardtii*, a typically bacterial enzyme in a eukaryotic alga. Eukaryotic Cell 7, 518–526. 18245276 10.1128/EC.00368-07 PMC2268514

[CIT0026] Hoefnagel MH Atkin OK Wiskich JT 1998 Interdependence between chloroplasts and mitochondria in the light and the dark. Biochimica et Biophysica Acta - Bioenergetics 1366, 235–255.

[CIT0027] Holzer H Goedde HW 1957 Two ways from pyruvate to acetyl-coenzyme A in yeast. Biochemische Zeitschrift 329, 175–191. 13522696

[CIT0028] Ke J Behal RH Back SL Nikolau BJ Wurtele ES Oliver DJ 2000 The role of pyruvate dehydrogenase and acetyl-coenzyme A synthetase in fatty acid synthesis in developing Arabidopsis seeds. Plant Physiology 123, 497–508. 10859180 10.1104/pp.123.2.497 PMC59018

[CIT0029] Li J Han D Wang D 2014 Choreography of transcriptomes and lipidomes of Nannochloropsis reveals the mechanisms of oil synthesis in microalgae. Plant Cell 26, 1645–1665. 24692423 10.1105/tpc.113.121418 PMC4036577

[CIT0030] Lin M Oliver DJ 2008 The role of acetyl-coenzyme a synthetase in Arabidopsis. Plant Physiology 147, 1822–1829. 18552233 10.1104/pp.108.121269 PMC2492652

[CIT0031] Linka M Weber APM 2005 Shuffling ammonia between mitochondria and plastids during photorespiration. Trends in Plant Science 10, 461–465. 16143558 10.1016/j.tplants.2005.08.002

[CIT0032] Ma Z Chu C-H Cheng D 2009 A novel direct homogeneous assay for ATP citrate lyase. Journal of Lipid Research 50, 2131–2135. 19286649 10.1194/jlr.D900008-JLR200 PMC2739766

[CIT0033] Mellema S Eichenberger W Rawyler A Suter M Tadege M Kuhlemeier C 2002 The ethanolic fermentation pathway supports respiration and lipid biosynthesis in tobacco pollen. Plant Journal of Cellular and Molecular Biology 30, 329–336. 10.1046/j.1365-313x.2002.01293.x 12000680

[CIT0034] Miller R Wu G Deshpande RR 2010 Changes in transcript abundance in *Chlamydomonas reinhardtii* following nitrogen deprivation predict diversion of metabolism. Plant Physiol . 154, 1737–1752. 20935180 10.1104/pp.110.165159 PMC2996024

[CIT0035] Ngan CY Wong CH Choi C 2015 Lineage-specific chromatin signatures reveal a regulator of lipid metabolism in microalgae. Nature Plants DOI: 10.1038/NPLANTS.2015.107. 10.1038/nplants.2015.107 27250540

[CIT0036] Nunes-Nesi A Fernie AR Stitt M 2010 Metabolic and signaling aspects underpinning the regulation of plant carbon nitrogen interactions. Molecular Plant 3, 973–996. 20926550 10.1093/mp/ssq049

[CIT0037] Oa ND Bo C 2005 Metabolite profiling of *Chlamydomonas reinhardtii* under nutrient deprivation. Plant Physiology 139, 1995–2005. 16306140 10.1104/pp.105.071589 PMC1310576

[CIT0038] Otterstedt K Larsson C Bill RM Stahlberg A Boles E Hohmann S Gustafasson L 2004 Switching the mode of metabolism in the yeast *Saccharomyces cerevisiae* . EMBO Report 5, 532–537. 10.1038/sj.embor.7400132 PMC1299050 15071495

[CIT0039] Padmasree K Padmavathi L Raghavendra AS 2002 Essentiality of mitochondrial oxidative metabolism for photosynthesis: optimization of carbon assimilation and protection against photoinhibition. Critical Reviews in Biochemical and Molecular Biology 37, 71–119. 10.1080/10409230290771465 12027265

[CIT0040] Porra RJ 2002 The chequered history of the development and use of simultaneous equations for the accurate determination of chlorophylls a and b. Photosynthesis Research 73, 149–156. 16245116 10.1023/A:1020470224740

[CIT0041] Praveenkumar R Shameera K Mahalakshmi G Akbarsha MA Thajuddin N 2012 Influence of nutrient deprivations on lipid accumulation in a dominant indigenous microalga *Chlorella sp*., BUM11008: Evaluation for biodiesel production. Biomass and Bioenergy 37, 60–66.

[CIT0042] Pronk JT Yde Steensma H Van Dijken JP 1996 Pyruvate metabolism in *Saccharomyces cerevisiae* . Yeast 12, 1607–1633. 9123965 10.1002/(sici)1097-0061(199612)12:16<1607::aid-yea70>3.0.co;2-4

[CIT0043] Rachutin-Zalogin T Pick U 2014 *a* Azide improves triglyceride yield in microalgae. Algal Research 3, 8–16.

[CIT0044] Rachutin-Zalogin T Pick U 2014 *b* . Inhibition of nitrate reductase by azide in microalgae results in triglycerides accumulation. Algal Research 3, 17–23.

[CIT0045] Raghavendra AS Padmasree K 2003 Beneficial interactions of mitochondrial metabolism with photosynthetic carbon assimilation. Trends in Plant Science 8, 546–53. 14607100 10.1016/j.tplants.2003.09.015

[CIT0046] Ramanan R Kim B-H Cho D-H Ko S-R Oh H-M Kim H-S 2013 Lipid droplet synthesis is limited by acetate availability in starchless mutant of *Chlamydomonas reinhardtii* . FEBS letters 587, 370–377. 23313852 10.1016/j.febslet.2012.12.020

[CIT0047] Ratledge C Wynn JP 2002 The biochemistry and molecular biology of lipid accumulation in oleaginous microorganisms. Advances in Applied Microbiology 51, 1–51. 12236054 10.1016/s0065-2164(02)51000-5

[CIT0048] Rismani-Yazdi H Haznedaroglu BZ Bibby K Peccia J 2011 Transcriptome sequencing and annotation of the microalgae *Dunaliella tertiolecta*: pathway description and gene discovery for production of next-generation biofuels. BMC Genomics 12, 148. 21401935 10.1186/1471-2164-12-148 PMC3061936

[CIT0049] Rodolfi L Chini Zittelli G Bassi N Padovani G Biondi N Bonini G Tredici MR 2009 Microalgae for oil: strain selection, induction of lipid synthesis and outdoor mass cultivation in a low-cost photobioreactor. Biotechnology and Bioenergy 102, 100–112. 10.1002/bit.22033 18683258

[CIT0050] Roughan G 1994 A semi-preparative enzymic synthesis of malonyl-CoA from [^14^C]acetate and ^14^CO_2_: labelling in the 1, 2 or 3 position. Biochemical Journal 300, 355–358. 8002939 10.1042/bj3000355 PMC1138169

[CIT0051] Roughan PG Ohlrogge JB 1996 Evidence that isolated chloroplasts contain an integrated lipid-synthesizing assembly that channels acetate into long-chain fatty acids. Plant Physiology 110, 1239–1247. 12226255 10.1104/pp.110.4.1239 PMC160916

[CIT0052] Sasaki Y Nagano Y 2004 Plant acetyl-CoA carboxylase: structure, biosynthesis, regulation, and gene manipulation for plant breeding. Bioscience Biotechnology and Biochemistry 68, 1175–1184. 10.1271/bbb.68.1175 15215578

[CIT0053] Schmollinger S Mühlhaus T Boyle NR 2014 Nitrogen-sparing mechanisms in Chlamydomonas affect the transcriptome, the proteome and photosynthetic metabolism. Plant Cell 26, 1410–1435. 24748044 10.1105/tpc.113.122523 PMC4036562

[CIT0054] Schuhmann H Lim DK Schenk PM 2014 Perspectives on metabolic engineering for increased lipid contents in microalgae. Biofuels 3, 71–86.

[CIT0055] Shiao T Ellis MH Dolferus R Dennis ES Doran PM 2002 Overexpression of alcohol dehydrogenase or pyruvate decarboxylase improves growth of hairy roots at reduced oxygen concentrations. Biotechnology and Bioenergy 77, 455–461. 10.1002/bit.10147 11787018

[CIT0056] Shtaida N Khozin-Goldberg I Solovchenko A Chekanov K Didi-Cohen S Leu S Cohen Z Boussiba S 2014 Downregulation of a putative plastid PDC E1α subunit impairs photosynthetic activity and triacylglycerol accumulation in nitrogen-starved photoautotrophic *Chlamydomonas reinhardtii* . J Experimental Botany 65, 6563–6576. 10.1093/jxb/eru374 PMC4246187 25210079

[CIT0057] Siaut M Cuiné S Cagnon C 2011 Oil accumulation in the model green alga *Chlamydomonas reinhardtii*: characterization, variability between common laboratory strains and relationship with starch reserves. BMC Biotechnology 11, 7. 21255402 10.1186/1472-6750-11-7 PMC3036615

[CIT0058] Slabas AR White A Ohara P Fawcett T 2002 Investigations into the regulation of lipid biosynthesis in *Brassica napus* using antisense down-regulation. Biochemical Society Transactions 30, 56–59. 10.1042/bst0301056 12440971

[CIT0059] Tadege M Kuhlemeier C 1997 Aerobic fermentation during tobacco pollen development. Plant Molecelular Biology 35, 343–354. 10.1023/a:1005837112653 9349258

[CIT0060] Terashima M Specht M Naumann B Hippler M 2010 Characterizing the anaerobic response of *Chlamydomonas reinhardtii* by quantitative proteomics. Molecular and Cellular Proteomics 9, 1514–1532. 20190198 10.1074/mcp.M900421-MCP200 PMC2938099

[CIT0061] Tokuyasu KT 1973 A technique for ultracryotomy of cell suspensions and tissues. The Journal of Cell Biology 57, 551–65. 4121290 10.1083/jcb.57.2.551 PMC2108989

[CIT0062] Tovar-Mendez A Miernyk JA Randall DD 2003 Regulation of pyruvate dehydrogenase complex activity in plant cells. European Journal of Biochemistry 270, 1043–1049. 12631264 10.1046/j.1432-1033.2003.03469.x

[CIT0063] Wellen KE Hatzivassiliou G Sachdeva UM Bui T V Cross JR Thompson CB 2009 ATP-citrate lyase links cellular metabolism to histone acetylation. Science 324, 1076–1080. 19461003 10.1126/science.1164097 PMC2746744

[CIT0064] Yeh K-L Chang J-S 2011 Nitrogen starvation strategies and photobioreactor design for enhancing lipid content and lipid production of a newly isolated microalga *Chlorella vulgaris* ESP-31: implications for biofuels. Biotechnology Journal 6, 1358–1366. 21381209 10.1002/biot.201000433

[CIT0065] Zabalza A van Dongen JT Froehlich A 2009 Regulation of respiration and fermentation to control the plant internal oxygen concentration. Plant Physiology 149, 1087–1098. 19098094 10.1104/pp.108.129288 PMC2633817

