# Supplementary material for: Acetyl-CoA synthetase is activated as part of the PDH-bypass in the oleaginous green alga Chlorella desiccata
Source: J Exp Bot. 2015 Sep 10;66(22):7287–98. doi: 10.1093/jxb/erv424 (PMC4765794; doi:10.1093/jxb/erv424)
Supplement: Supplementary Data [file supp_66_22_7287__index.html]

Acetyl-CoA synthetase is activated as part of the PDH-bypass in the oleaginous green alga Chlorella desiccata — Acetyl-CoA synthetase is activated as part of the PDH-bypass in the oleaginous green alga Chlorella desiccata — Supplementary Data 

# Acetyl-CoA synthetase is activated as part of the PDH-bypass in the oleaginous green alga *Chlorella desiccata*

## Supplementary Data

Data files

- Supplementary Data - Supplementary Data
