# Supplementary material for: Acetyl-CoA synthetase is activated as part of the PDH-bypass in the oleaginous green alga Chlorella desiccata
Source: J Exp Bot. 2015 Sep 10;66(22):7287–98. doi: 10.1093/jxb/erv424 (PMC4765794; doi:10.1093/jxb/erv424)
Supplement: Supplementary Data [file supp_erv424_jexbot151381_file001.pdf]

Acetyl-CoA synthetase is activated as part of the PDH-bypass in the oleaginous green alga *Chlorella desiccata*

Omri Avidan* and Uri Pick*;

A

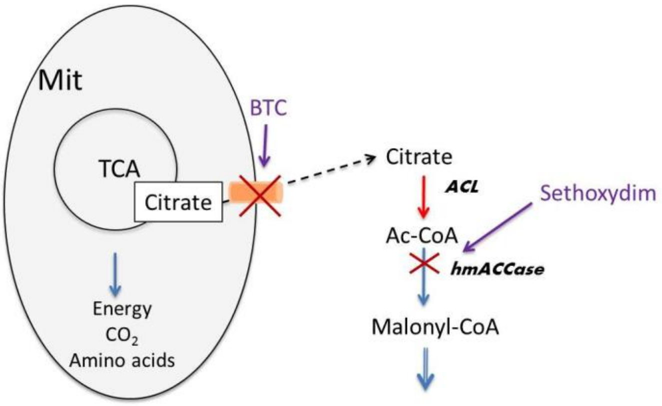

B

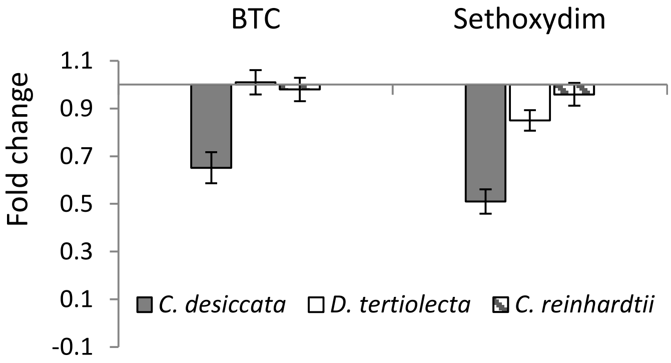

Supplementary 1: Effects of citrate efflux and hmACCase inhibitors on TAG level. Cultures of *C. desiccata* and of *D. tertiolecta* were induced in the presence or absence of BTC and sethoxydim. A, schematic illustration if inhibition sites of BTC and sethoxydim. B, relative changes in TAG levels compared to control N deprived cells (means ± SD of 3 independent experiments).
